# Ferroportin disease: A systematic meta-analysis of clinical and molecular findings^☆^

**DOI:** 10.1016/j.jhep.2010.05.016

**Published:** 2010-11

**Authors:** Roman Mayr, Andreas R. Janecke, Melanie Schranz, William J.H. Griffiths, Wolfgang Vogel, Antonello Pietrangelo, Heinz Zoller

**Affiliations:** 1 Department of Medicine II, Gastroenterology and Hepatology, Medical University of Innsbruck, Austria; 2 Department of Pediatrics II, Medical University of Innsbruck, Austria; 3 Department of Hepatology, Cambridge University Hospitals, NHS Foundation Trust, Cambridge, UK; 4 Department of Medicine, University Hospital of Modena, Policlinico di Modena, Modena, Italy

**Keywords:** AUROC, area under the receiver operator curve, ER, endoplasmic reticulum, HIC, hepatic iron concentration, JAK2, Janus Kinase 2, MRI, magnetic resonance imaging, PolyPhen, polymorphism phenotyping, ROC, receiver operator curve, SIFT, sorting intolerant from tolerant, SLC40A1, Hemochromatosis type IV, Iron, Bio-informatics, SLC11A3, IREG1, FPN1, PolyPhen

## Abstract

**Background & Aims:**

*Classical* ferroportin disease is characterized by hyperferritinemia, normal transferrin saturation, and iron overload in macrophages. A *non-classical* form is characterized by additional hepatocellular iron deposits and a high transferrin saturation. Both forms demonstrate autosomal dominant transmission and are associated with ferroportin gene (*SLC40A1*) mutations. *SLC40A1* encodes a cellular iron exporter expressed in macrophages, enterocytes, and hepatocytes. The aim of the analysis is to determine the penetrance of *SLC40A1* mutations and to evaluate *in silico* tools to predict the functional impairment of ferroportin mutations as an alternative to *in vitro* studies.

**Methods:**

We conducted a systematic review of the literature and meta-analysis of the biochemical presentation, genetics, and pathology of ferroportin disease.

**Results:**

Of the 176 individuals reported with *SLC40A1* mutations, 80 were classified as *classical* phenotype with hyperferritinemia and normal transferrin saturation. The *non-classical* phenotype with hyperferritinemia and elevated transferrin saturation was present in 53 patients. The remaining patients had normal serum ferritin or the data were reported incompletely. Despite an increased hepatic iron concentration in all biopsied patients, significant fibrosis or cirrhosis was present in only 11%. Hyperferritinemia was present in 86% of individuals with ferroportin mutations. Bio-informatic analysis of ferroportin mutations showed that the PolyPhen score has a sensitivity of 99% and a specificity of 67% for the discrimination between ferroportin mutations and polymorphisms.

**Conclusions:**

In contrast to HFE hemochromatosis, ferroportin disease has a high penetrance, is genetically heterogeneous and is rarely associated with fibrosis. *Non-classical* ferroportin disease is associated with a higher risk of fibrosis and a more severe overload of hepatic iron.

## Introduction

Ferroportin disease is a clinically and genetically heterogeneous iron overload syndrome [1]. Its clinical presentation has been documented in individual case reports and small case series. The natural course has been reported in one small series [2]. Hyperferritinemia, a normal to low transferrin saturation and Kupffer cell iron storage, presenting as hepatic and spleen iron overload, are considered characteristic features of *classical* ferroportin disease [3]. Increased transferrin saturation and hepatocellular iron overload, in addition to hyperferritinemia and macrophage iron loading, is considered characteristic for the *non-classical* phenotype [3,4].

A genotype–phenotype correlation was suggested to explain the clinical heterogeneity of the disease where most mutations (e.g. A77D, D157G, V162del, N174I, Q182H, Q248H, and G323V) are associated with *classical* ferroportin disease [5–8]. Distinct mutations (e.g. N144H, Y64N, C326Y/S, S338R, Y501C) have been found in patients who presented with the *non-classical* phenotype [5,9–12].

Ferroportin is the only known mammalian iron exporter and is expressed in macrophages and the basolateral membrane of enterocytes and hepatocytes [13–15]. In *classical* ferroportin disease, macrophage iron overload results from cellular iron export deficiency, i.e. loss of ferroportin function [6]. The D157G mutation leads to hepcidin-independent, constitutive binding of Janus Kinase 2 (JAK2) and thus triggers ferroportin down-regulation [16]. The concept that other loss of function mutations of ferroportin result in intracellular retention of the iron export pump has been challenged recently [17,18], but alternative mechanisms leading to iron transport deficiency have not been fully elucidated.

Ferroportin inactivation is mediated by the peptide hormone hepcidin [19,20]. Distinct mutations render ferroportin resistant to inactivation by hepcidin and cause a gain of iron export function, which results in hyper-absorption of dietary iron.

Hepcidin is secreted by hepatocytes into the circulation in response to pro-inflammatory cytokines, hepatocellular iron loading, and endoplasmic reticulum stress [21,22]. Circulating hepcidin directly interacts with ferroportin and induces its internalization and degradation [23]. The free thiol group of cysteine 326 of ferroportin is essential for hepcidin binding and ferroportin gene mutations affecting residue 326 result in hepcidin resistance [7,24]. Patients with ferroportin disease due to hepcidin resistance mutations, exhibit a *non-classical* phenotype [8,25,26].

Functional studies in cells overexpressing ferroportin variants have been used to differentiate between gain-of-function and loss-of-function mutations and benign sequence variants. The functional relevance of ferroportin mutations can also be deduced from mutation segregation studies within families with ferroportin disease. Association studies of *SLC40A1* polymorphisms with serum iron indices in population studies indirectly provide evidence for their functional relevance [27–29].

Bio-informatic tools were used to complement the genetic and functional studies. SIFT [30] and PolyPhen [31] provide an *in silico* prediction of the functional consequences of missense mutations. The use of these tools in differentiating between diseases-associated ferroportin mutations from benign sequence variants was assessed. The clinical presentation and penetrance of ferroportin gene mutations were assessed from a systematic review of the literature.

## Methods

### Search strategy

A systematic literature search was undertaken in Medline from 1996 to June 2009 according to the following search strategy: #1 (‘*SLC40A1*’ or ‘SLC11A3’ or ‘ferroportin’ or ‘IREG’ or ‘IREG1’ or ‘IREG-1’ or ‘ferroportin’ or ‘ferroportin1’ or ‘ferroportin-1’ or ‘FPN’ or ‘FPN1’ or ‘FPN-1’).mp. [mp = title, original title, abstract, name of substance word, subject heading word]; #2 (‘mutation’ or ‘variant’).mp. [mp = title, original title, abstract, name of substance word, subject heading word]; # 3: #1 and #2. This search retrieved 143 references and was supplemented by manual searching for references of the relevant articles identified. After exclusion of review articles and non-human studies, data from 49 studies were included. This search was complemented by an Embase search using the search strategy: #1 (?*SLC40A1*? or ?SLC11A3? or ?ferroportin? or ?IREG? or ?IREG1? or ?IREG-1? or ?ferroportin? or ?ferroportin1? or ?ferroportin-1? or ?FPN? or ?FPN1? or ?FPN-1?).mp. [mp = title, original title, abstract, name of substance word, subject heading word]; #2 (?mutation? or ?variant?).mp. [mp = title, original title, abstract, name of substance word, subject heading word]; # 3: #1 and #2.

### Prediction of the functional effect of amino acid substitutions by *in silico* analysis

Two algorithms were used to evaluate the impact of amino acid substitutions on the function of ferroportin: polymorphism phenotyping (PolyPhen) [31–33] and sorting intolerant from tolerant (SIFT) [30]. Genetic variants of SLC40A1, identified in patients with suspected iron overload, were collected from the systematic review of the literature. In addition, single nucleotide polymorphisms of SLC40A1 were collected from the literature and from ENSEMBL (http://www.ensembl.org/) and the NCBI databases (http://www.ncbi.nlm.nih.gov/). A SLC40A1 variant was considered a mutation in case its frequency in the control population was undetermined, or lower than 1:50, and the variant was not reported in a SNP database [27–29,34,35].

### PolyPhen

PolyPhen (= *Poly*morphism *Phen*otyping) is an automatic tool for prediction of possible impact of an amino acid substitution on the structure and function of a human protein. The predictive value of the PolyPhen score was demonstrated in *diabetes mellitus* [36] and cancer genetics [37]. This prediction is based on empirical rules which are applied to the sequence, phylogenetic, and structural information characterizing the substitution [31]. PolyPhen was employed, as available at http://coot.embl.de/PolyPhen/ (version August 12, 2008), using the NCBI protein accession number Q9NP59 (*SLC40A1*) for ferroportin. In all cases, the prediction basis was only the default position-specific independent counts (PSIC) score derived from multiple sequence alignment of observations. PolyPhen scores of >2.0 indicated protein function as ‘probably damaging’ and scores of <1.5 as ‘benign’. A PolyPhen score of 1.5–2.0 is classified as ‘possibly’ damaging.

A phylogenetic sequence alignment of selected ferroportin protein sequences is shown in Fig. 2.

### SIFT

SIFT (= Sorting Intolerant from Tolerant) is a sequence-homology-based tool that presumes that important amino acids will be conserved in the protein family. Changes at well-conserved positions tend to be predicted as deleterious. SIFT is a multi-step procedure that uses multiple alignment information to predict tolerated and deleterious substitutions for every position of the query sequence [30]. The SIFT Blink algorithm was applied to compare amino acid sequences of different organisms chosen from precomputed NCBI BLAST searches. For query in SIFT Blink, GI: 49065554 (*SLC40A1*) was used to analyze the protein sequence by using the parameter ‘best BLAST hit to each organism’ and omitting sequences >90% identical to query. Edited alignments were reanalyzed by the appropriate module of SIFT (http://blocks.fhcrc.org/sift/SIFT_aligned_seqs_submit.html). Results were reported as ‘affects protein function’ or ‘tolerated’ according to this analysis.

To evaluate the bio-informatic tools to distinguish between benign and disease sequence variants, the SLC40A1 polymorphisms were used as true negatives and genetic variants of SLC40A1 identified in patients with ferroportin disease as true positives. The PolyPhen and SIFT scores were then analyzed in a ROC curve for their sensitivity and specificity.

### Statistical analysis

For statistical analysis the software package for social sciences (SPSS v15.0) was used. To test for correlation between parameters, Pearson correlation was carried out for normally distributed parameters and Spearman rank correlation for non-normally distributed parameters. Normality of distribution was tested by Kolmogorov–Smirnov curve-fitting. Differences between groups were analyzed by Kruskal Wallis test for non-Gaussian distributed variables. Correlations and differences were considered significant if *p* <0.05. Sensitivity and specificity of SIFT and PolyPhen scores were calculated form the ROC curve analysis using mutations identified in patients with ferroportin disease as true positives and polymorphisms reported in the SNP database (http://www.ncbi.nlm.nih.gov/sites/entrez) as true negatives.

## Results

### High biochemical penetrance and variable clinical presentation of SLC40A1 mutations

One hundred and seventy-six individuals with ferroportin gene mutations were reported in the literature, and their demographic and biochemical data are listed in Table 1. The diagnosis of ferroportin disease was most frequently made in the 3rd to 4th decade of life and 43% of patients were female.

To describe the disease presentation and the penetrance of underlying mutations, serum iron parameters, liver biopsy findings, MRI, comorbidities, and phlebotomy status were assessed. These were reported incompletely for several patients (Table 1).

Forty-five comorbidities were reported in 29 patients (viral hepatitis 3, steatosis 12, obesity 5, diabetes 8, impaired glucose tolerance 2, hyperlipidemia 2, increased alcohol consumption 2, and sarcoidosis 2). Mean age and mean serum ferritin were significantly higher in patients with comorbidities, than in patients without comorbidities, (52 ± 15 years vs. 39 ± 20 years *p *= 0.002 and 3133 ± 3270 μg/L vs. 1965 ± 2067 μg/L, *p *= 0.017). Fibrosis ⩾F1 (Metavir) was significantly more common in patients with comorbidities than in patients without comorbidities (12 of 44 vs. 10 of 18; Fisher’s exact test *p* <0.05).

The biochemical penetrance of ferroportin disease was 86% (142 of 166 patients), defined as hyperferritinemia >300 μg/L in males and >200 μg/L in females. We found a significant correlation between serum ferritin and age (*r*^2^ = 0.344, *n* = 158, *p* <0.001). The phenotype of hyperferritinemic patients was classified as *classical* if transferrin saturation was normal and as *non-classical* if transferrin saturation was increased (males >50%, females >45%). *Classical* disease was present in 80 patients as opposed to 53 patients with *non-classical* disease, whose mean age at presentation was significantly higher (44 vs. 37 years, *p* <0.05) (Table 2). Serum iron parameters for the remaining 43 patients were either incompletely reported or normal (16 patients). Differences between *classical* and *non-classical* phenotype are evident in Table 2 where mean hemoglobin, ferritin, and hepatic iron concentration (HIC) were significantly lower in the *classical* disease cohort.

Liver iron was increased in 30 of 31 reported patients, with a mean HIC of 310 μmol/g (normal <25 μmol/g) [38]. Liver fibrosis ⩾F2 or liver cirrhosis was present in 7 of 53 biopsied patients (13%), and correlated with age (mean 53.3 vs. 36.2 years, *p* <0.001). Cirrhosis was reported in 4 of 176 patients, of whom 2 had the N144H mutation, one patient the C326S and one the I180T mutation [25,39,40]. One patient with ferroportin disease was diagnosed with hepatocellular carcinoma which was possibly linked with occult hepatitis B virus infection [2].

In search of factors that discriminate ferroportin disease severity, the correlation between patient demographics, serum iron parameters, and liver biopsy findings was determined. The stage of liver fibrosis correlated significantly with age (*r* = 0.54, *n* = 53, *p* <0.001), but neither with HIC nor with serum ferritin. HIC and transferrin saturation (*r* = 0.528, *p *= 0.003, *n* = 29), and HIC and serum ferritin (*r* = 0.423, *p *= 0.018, *n* = 31) showed a moderate correlation.

### Genotype–phenotype correlation partly explains variability in clinical presentation

**Fig. 1:**
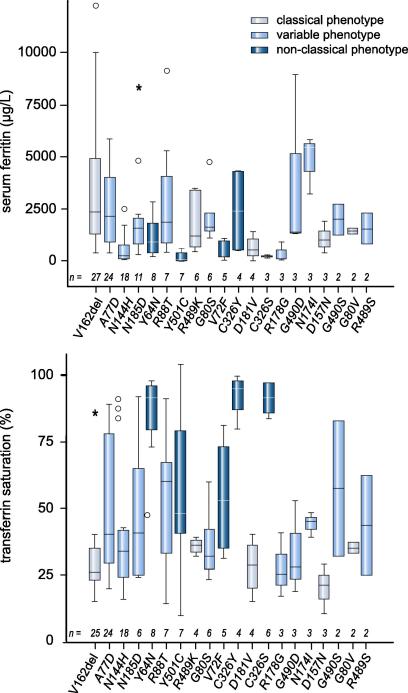
**Genotype to phenotype correlation of ferroportin disease: patients with ferroportin disease identified from the systematic meta-analysis were grouped according to the *SLC40A1* mutation.** Box-Whisker blots of (A) serum ferritin, and (B) transferrin saturation in mutations reported in more than 5 patients are shown. Boxes represent 25th and 75th percentile, Whiskers range and horizontal lines represent the median. Outliers are shown as circles. Grey, dark blue, and light blue boxes indicate that all patients reported with the respective mutation were classified as *classical*, *non-classical,* or variable biochemical phenotype, respectively, i.e. all patients had low or normal transferrin saturation (grey), increased transferrin saturation (dark blue), or different patients with the same mutation had variable transferrin saturation (light blue). Any outliers are marked with a circle and extreme cases with an asteriks.

**Fig. 2:**
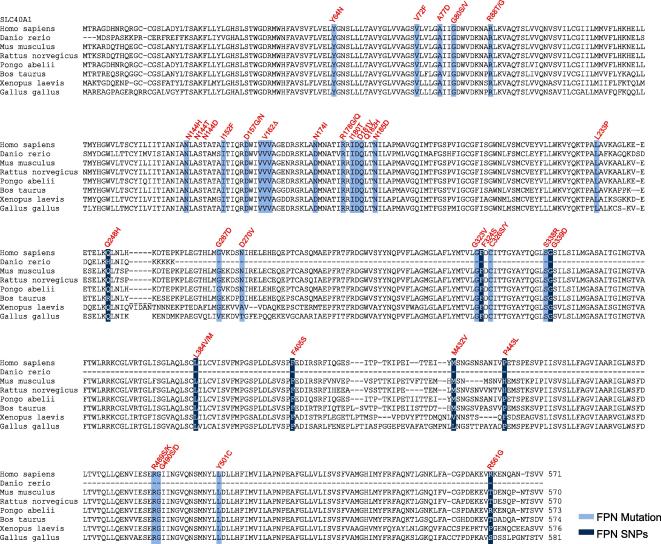
Phylogenetic alignment of ferroportin protein sequences of various species: Disease-associated ferroportin gene mutations are highlighted in light blue. Single nucleotide polymorphisms are shown in dark blue.

Of 31 *SLC40A1* mutations, 6 and 5 were unequivocally associated with the *classical* or the *non-classical* phenotype, respectively (Table 2). Variable phenotypes in individual patients with the same mutation were reported for 9 mutations (Fig. 1 and Table 3).

MRI findings of liver and spleen iron were reported for 8 ferroportin mutations (c.-188A>G, G490D, I152F, L233P, R178G, V162del, Y64 N, Y501C) [12,41–45,5,46] where the small number of patients prohibits assessment of the diagnostic performance of MRI for the classification of ferroportin disease.

### How to differentiate between SLC40A1 mutations and polymorphisms

Ferroportin disease is genetically heterogeneous with 36 different *SLC40A1* mutations reported [8–10,12,26–29,38–41,45–68] as summarized in Table 3. In addition, 9 ferroportin gene polymorphisms were reported, 3 of which were associated with increased serum ferritin in various populations (Table 4) [29,34]. The allele frequencies of 29 disease-associated ferroportin mutations had been determined in matched populations and was found to be <1:100. It remains unclear whether L233V and D270P, each of which have been identified in single patients with iron overload, are disease-causing mutations or represent benign sequence variants [43,65].

**Fig. 3:**
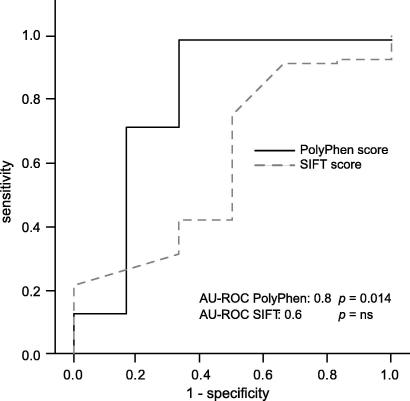
**Diagnostic performance analysis (ROC curve) of PolyPhen and SIFT scores for the prediction of the functional consequence of ferroportin gene variants.** Genetic variants of *SLC40A1* reported as polymorphisms were used as true negatives and genetic variants of *SLC40A1* identified in patients with ferroportin disease were used as true positives. ROC curves are shown with the sensitivity plotted along the abscissa, and 1 – specificity plotted along the ordinate.

To determine whether bio-informatic tools PolyPhen and SIFT [30,32,69] can discriminate between disease-causing *SLC40A1* mutations and benign sequence variants, their ability to classify known ferroportin sequence variants accordingly was tested. The predicted functional impairment of individual sequence variants from the PolyPhen and SIFT tools were used as outcome variables for a receiver-operator curve analysis. The PolyPhen score had a high diagnostic accuracy with a calculated area under the ROC curve (AUROC) of 0.798 (*p *= 0.014) (Fig. 3). Using the descriptive output of PolyPhen (i.e. probably damaging, possibly damaging or benign) as predictors of the functional consequence of particular gene variants, the sensitivity of the PolyPhen score was 99% and the specificity 67% (Table 2 and Fig. 3). SIFT analysis predicted that 23 of 32 disease associated missense mutations ‘affects’ protein function and 9 are ‘tolerated’. Of 9 reported polymorphisms 7 were classified as ‘tolerated’ (AUROC 0.605 *p *= 0.37). The bio-informatics PolyPhen tool has a higher sensitivity and specificity than the SIFT score for the discrimination between missense mutations and single nucleotide polymorphisms of *SLC40A1*.

Serum ferritin concentration (*r* = 0.226, *n* = 136, *p *= 0.008), and transferrin saturation (*r* = 0.198, *n* = 128, *p *= 0.025) were correlated with the PolyPhen score, suggesting that ferroportin disease is more severe when phylogenetically more conserved residues are affected by the mutation.

## Discussion

Ferroportin disease is the commonest genetic iron overload syndrome after HFE associated hemochromatosis and has a variable clinical presentation. This review of the literature and meta-analysis shows that hyperferritinemia and increased hepatic iron concentration are characteristic signs of the disease. In contrast, elevated transferrin saturation and splenic iron overload are variably present. Hyperferritinemia was found in 86% of individual with ferroportin mutations, but incomplete reporting is a limitation for the accurate assessment of disease penetrance. The observed correlation between age and serum ferritin supports the concept that ferroportin disease is a progressive iron overload disorder. It is therefore possible that patients detected through cross-sectional study designs have not yet had time to develop the ‘full-blown’ disease. Hence, it is difficult to assess the true penetrance without further follow-up data. By comparison, in genetic hemochromatosis hyperferritinemia was present in 55–86% of *C282Y* homozygotes [70,71] and cirrhosis was present in 36% of more severely affected *C282Y* homozygous individuals with ferritin >1000 μg/L [72].

To assess the pathology of ferroportin disease, the findings of liver biopsies were reviewed. However, liver biopsies were performed in only a few patients and the results were inconsistently reported. This prohibits a systematic classification of ferroportin disease based on liver biopsy results. In patients who underwent liver biopsies for ferroportin disease, hepatic iron concentration was invariably increased and the penetrance of liver fibrosis ⩾F2 was 13%. Age, hepatic iron concentration, and comorbidities were associated with fibrosis stage and are thus potential risk factors for fibrosis in ferroportin disease.

A simple biochemical classification strategy based on transferrin saturation was used as an alternative to liver biopsy. Transferrin saturation is low to normal in *classical* ferroportin disease and increased in patients with the *non-classical* phenotype. An arbitrary threshold for transferrin saturation of 45% for females and 50% for males was used to distinguish *classical* from the *non-classical* phenotype. The value of this simple classification is supported by our meta-analysis, which shows that patients with the *classical* phenotype have lower hemoglobin concentrations. This may explain why patients with *classical* ferroportin disease appear to tolerate therapeutic venesection less well than hemochromatosis patients. MRI was proposed as an alternative to liver biopsy to discriminate between *classical* and *non-classical* ferroportin disease. However, no correlation between parameters of disease severity and MRI findings has been reported [73].

The genotype to phenotype correlation suggests that ferroportin disease has a multifactorial cause because not all mutations were unambiguously correlated with the *classical* or *non-classical* phenotype in all reported patients with a particular mutation. Our meta-analysis suggests that Y64N, V72F, and Y501C confer hepcidin resistance because the phenotypic presentation as *non-classical* disease is similar to the C326Y mutation [7,74,75]. Similarly, *classical* mutations D157N, D181V, G80V, Q182H, R489K, and V162del may impair the iron export function of ferroportin through incorrect folding, subcellular mislocalization, or direct inhibition of the iron transport function [6,18,75].

We have assessed PolyPhen and SIFT [76] as alternatives to predict the effect of ferroportin mutations on protein function, because functional studies of ferroportin mutants [6,17,18,68,75] have shown conflicting results [17,18]. Our analysis shows that PolyPhen is a sensitive tool to identify disease-causing gene variants by classifying mutations identified in patients with iron overload as ‘possibly’ or ‘probably’ damaging. The sensitivity of the PolyPhen score is 99% and the specificity 67%. These findings suggest that the functional effect of ferroportin mutations can be predicted *in silico* and that these tools may help to interpret the relevance of newly-identified mutations in the future.

The systematic review of the molecular genetics and the clinical presentation of ferroportin disease highlights the heterogeneity of the disease and the difficulties in securing the diagnosis. Similar to *HFE* hemochromatatosis, age, and comorbidities appear to be important risk factors for the development of fibrosis in patients with ferroportin disease [77]. PolyPhen helps to score newly-identified, presumed *SLC40A1* mutations; identification of a genetic variant in *SLC40A1* is not sufficient to confirm ferroportin disease in patients with hyperferritinemia.

## Conflict of interest

The authors who have taken part in this study declared that they do not have anything to disclose regarding funding or conflict of interest with respect to this manuscript.

## Figures and Tables

**Table 1:** **Summary of demographics and clinical characteristics of 176 patients with ferroportin disease**. (Note not all parameters were reported for each patient.)

∗Phlebotomy was reported for 82 patients. Grams iron removed was reported for 20 patients.

**Table 2:** **Characteristics of *classical* vs. *non-classical* phenotype of ferroportin disease as defined by the presence of hyperferritinemia with normal transferrin saturation (*classical*) versus patients with hyperferritinemia and elevated transferrin saturation (*non-classical*)**. Data are shown as means ± standard deviation. Differences were tested for significance using student’s *t*-test.

**Table 3:** **Molecular genetics of *SLC40A1* mutations.** The frequency of mutations highlighted with ∗ in the control population can be inferred from studies, in which control populations have been screened for the presence of another mutation, affecting the same residue.

∗∗Predicted G468S.

**Table 4:** ***SLC40A1* non-synonymous single nucleotide polymorphisms.** SNPs, which have been associated with high serum iron parameters are highlighted in bold and italics.

^a^As reported in the NCBI SNP database (http://www.ncbi.nlm.nih.gov/projects/SNP/snp_ref.cgi?chooseRs=coding&go=Go&locusId=30061). ^b^http://www.ensembl.org/Homo_sapiens/Variation/Summary?v=ENSSNP12180430;vdb=variation.

## **Supplementary data**

Supplementary Table Results from histology, clinical and biochemical data of individual patients from whom a liver biopsy was reported.
