# Corrigendum to “Ferroportin disease: A systematic meta-analysis of clinical and molecular findings” J. Hepatol. 2010 Nov;53(5):941–949

**DOI:** 10.1016/j.jhep.2011.05.002

**Published:** 2011-09

**Authors:** Roman Mayr, Andreas R. Janecke, Melanie Schranz, William J.H. Griffiths, Wolfgang Vogel, Antonello Pietrangelo, Heinz Zoller

**Affiliations:** 1 Department of Medicine II, Gastroenterology and Hepatology Medical University of Innsbruck, Austria; 2 Department of Pediatrics II, Medical University of Innsbruck, Austria; 3 Department of Hepatology Cambridge University Hospitals NHS Foundation Trust, Cambridge, UK; 4 Department of Medicine, University Hospital of Modena, Policlinico di Modena, Modena, Italy

In the above named article there were three errors:

1. In the section on “How to differentiate between SLC40A1 mutations and polymorphisms”, a reference was left out of the following paragraph. The paragraph and reference are now listed below.
  Ferroportin disease is genetically heterogeneous with 36 different SLC40A1 mutations reported [8–10,12,26–29,38–41,45–68,78] as summarized in Table 3. In addition, nine ferroportin gene polymorphisms were reported, three of which were associated with increased serum ferritin in various populations (Table 4) [29,34]. The allele frequencies of 29 disease-associated ferroportin mutations had been determined in matched populations and was found to be <1:100. It remains unclear whether L233V and D270P, each of which have been identified in single patients with iron overload, are disease-causing mutations or represent benign sequence variants [43,65].

**Table 3:** **Molecular genetics of *SLC40A1* mutations**. The frequency of mutations highlighted with ∗ in the control population can be inferred from studies, in which control populations have been screened for the presence of another mutation, which affect the same residue.

**Table 4:** ***SLC40A1* non-synonymous single nucleotide polymorphisms**. SNPs, which have been associated with high serum iron parameters, are highlighted in bold and italics.
  [78] Wallace DF, Clark RM, Harley HA, Subramaniam VN. Autosomal dominant iron overload due to a novel mutation of ferroportin1 associated with parenchymal iron loading and cirrhosis. J Hepatol 2004;40:710–3.
2. In Table 3, Table 4 there were some errors. The tables are now reproduced correctly on the following pages.

The authors apologise for these errors.

