# Supplementary material for: Ferroportin disease: A systematic meta-analysis of clinical and molecular findings
Source: J Hepatol. 2010 Nov;53(5-3):941–9. doi: 10.1016/j.jhep.2010.05.016 (PMC2956830; doi:10.1016/j.jhep.2010.05.016)
Supplement: Supplementary Table — Results from histology, clinical and biochemical data of individual patients from whom a liver biopsy was reported. [file mmc1.pdf]

Reference	mutation (protein)	mutiation (cDNA level)	age	sex	Hb (g/dl)	ferritin (µg/L)	transferrin saturation (%)	iron removed	Fibrosis (ISAHK)	Cirrhosis	HII	HIC (µmol/g)	MRI	viral hepatitis	steatosis	BMI	metabloic syndrome	Ethanol (g/week)	other comorbidity	Biopsied	Histology M = Kupffer cells exclusively or prominent H = both or hepatocellular only
Subramaniam GUT 05	A77D	c.230C>A	45	m	12,2	3340	29	n.a.	0	0	2,1	96		no	no	normal	neg	20		Kupffer cell iron loading, min Hep	M
Lim BCMD 2008	A77D	c.230C>A	36	m		4000	45	yes	0			156,25		no	no		neg	270		prominent kupffer cell siderosis, grade 2 hep siderosis	M
Lim BCMD 2008	A77D	c.230C>A	34	m		4050	84	yes	1			388,4						40		prominent kupffer cell siderosis, grade 3 hep siderosis	M
Pietrangelo NEJM 99	A77D	c.230C>A	61	f	14,5	5846	89	12	1		5,1	310		no				<70		mixed: hep, reticuloendothelial	H
Pietrangelo NEJM 99	A77D	c.230C>A	59	m	16	5750	75	35	1		10,9	646		no				<70		mixed: hep, reticuloendothelial	H
Pietrangelo NEJM 99	A77D	c.230C>A	30	m	14,3	5600	88	16	1		27,2	815		no				<70		mixed: hep, reticuloendothelial	H
Pietrangelo NEJM 99	A77D	c.230C>A	34	m	15,7	2410	60	31	0		23,8	810		no				<70		mixed: hep, reticuloendothelial	H
Pietrangelo NEJM 99	A77D	c.230C>A	20	m	15	2700	40	12	0		11	220		no				<70		mixed: hep, reticuloendothelial	H
Pietrangelo NEJM 99	A77D	c.230C>A	21	f	14,2	890	30	6	0		5,2	110		no				<70		mixed: hep, reticuloendothelial	H
Pietrangelo NEJM 99	A77D	c.230C>A	37	m	15	3250	65	20	0		28,4	1050		no				<70		mixed: hep, reticuloendothelial	H
Pietrangelo NEJM 99	A77D	c.230C>A	14	m	13,8	815	28	4	0		5,4	75		no				<70		mixed: hep, reticuloendothelial	H
Liu Int Med 05		c.117A>G	43	f		9660	92	yes	0				liver/spleen	no			imp Glc Tol	0		mixed: hep&Kupff	H
Sham BCMD 05	C326S	c.977G>C	35	m				yes	0	1										marked Hep iron	H
Sham BCMD 05	C326S	c.977G>C	16	f			97	yes	0		2,3									marked Hep iron	H
Sham BCMD 05	C326S	c.977G>C	16	m			86	yes	0											marked Hep iron	H
Cemones BJH 05	D181V	c.846A>T	40	m		1400	40	yes	0											mixed: hep & kupffer	H
Lee ActHaem 2007	splice site (pred	c.1402G>A	59	m		1364	25	yes	0						mild		DM, Hyperlipid.	0		mixed: hep & kupffer	H
Jouanolle JHep 03	G490D	c.1468G>A	54	f	14,2	8943	53	yes	0		6,4	347	1							mixed: hep & kupffer	H
De Domenico Haemat 06	G80S	c.238G>A	34	m	15,8	4420	60	n.a.	0			954								yes	
Girelli JHep 08	I152F	c.758A>T	59	f		1771	22,7	yes	2				liver	HCV	mild	29	DM	no		Kupffer cell iron loading, min Hep	M
Girelli JHep 08	L233P	c.1012T>C	59	m		9000	74,8	yes	1				liver/spleen		grade 1		DM	140		Hepatocellular iron	H
Njajou BCMD 02	N144H	c.430A>G	76	n/a		219	40,4	no	0	1					yes			abuse		mixed	H
Njajou BCMD 02	N144H	c.430A>G	64	n/a		223	34,4	na	0	1						obesity				mixed	H
Njajou BCMD 02	N144H	c.430A>G	63	n/a		113	17	yes	1		27,5				yes					mixed	H
Njajou BCMD 02	N144H	c.430A>G	80	n/a		133	27,9	yes	1		12,3				yes					mixed	H
Njajou BCMD 02	N144H	c.430A>G	69	n/a		35	34	yes	1		12,8						DM			mixed	H
Njajou BCMD 02	N144H	c.430A>G	66	n/a				yes	1						yes		DM			mixed	H
Njajou BCMD 02	N144H	c.430A>G	53	n/a		31	24,1	yes	0							obesity				mixed	H
Njajou BCMD 02	N144H	c.430A>G	45	n/a		180	23,1	yes	1							obesity				mixed	H
Wallace JHep 04	N144D	c.430A>G	32	m		10510	99	yes	0		12,3	393	0					120	Sarcoidosis	Hepatocellular iron	H
Arden GUT 03	N144T	c.431A>C	48	m		2937	80	no	0		7,6	363								grade 4 hep iron, significant kupffer cell iron	M
De Domenico Haemat 06	N174I	c.521A>T	66	f	14,9	5815	48	n.a.	0			450								no hist info	
De Domenico Haemat 06	N174I	c.521A>T	38	f	13,4	5430	45	n.a.	0			650								no hist info	
Koyama Int Med 05	R489S	c.1467A>C	43	m		822	24,8	no	0					no			no	no		selective iron deposits in kupffer cells	M
Bach BCMD 06	R88T	c.263G>C	61	m		9075	91	16,5	1	0				no			no	mild		severe iron deposits in both	H
Bach BCMD 06	R88T	c.263G>C	56	m		2830	43	9,5	1	0		200								severe iron deposits in both	H
Bach BCMD 06	R88T	c.263G>C	53	m		5291	70	17,7	1	0				HBV/HCV						severe iron deposits in both	H
Bach BCMD 06	R88T	c.263G>C	23	m		1870	64	11	0											severe iron deposits in both	H
Wallace JHep 07	S338R	c.1014T>G	72	m		1990	90	yes	0			210	0		mild		imp. glc.tol	175		grade 4 iron in hep, kupff a periportal macros	H
Cazzola BJH 02	V162del	c.484_486del3	31	f	12	1396	15	no	0											selective iron deposits in kupffer cells	M
Devalia Blood 02	V162del	c.484_486del3	38	f		2855	40	yes	0											heavy iron deposits in both, kupffer and hepat	H
Devalia Blood 02	V162del	c.484_486del3	34	f		1150	31	yes	0				1, liver and spleen							marked iron accumulation in kupffer cells	M
Roetto Blood 02	V162del	c.484_486del3	26	f	11,5	1022	18	yes	0											positive especially in kupffer cells, with few hepatocyte granules	M
Wallace Blood 02	V162del	c.484_486del3	56	m		12000		yes	1		8,3	475								prominent kupffer cell loading in addition to hepatocell iron	M
Wallace Blood 02	V162del	c.484_486del3	73	m		10000	81	no	1											prominent kupffer cell loading in addition to hepatocell iron	M
Wallace Blood 02	V162del	c.484_486del3	20	m		1768	35	yes	1		5,2									prominent kupffer cell loading in addition to hepatocell iron	M
Wallace Blood 02	V162del	c.484_486del3	19	f		1182	19	yes	0		3,5									prominent kupffer cell loading in addition to hepatocell iron	M
Wallace GUT 05	V162del	c.484_486del3	36	f		3145	29	yes	0	0	9,1	316,1	1, liver				no			iron is prominetn in kupffer cells and hepatocytes	H
Zoller Hep 05	V162del	c.484_486del3	26	m	15,5	4935	23	no	0											predominantly in kupffer cells	M
Speletas BCMD 08	V162del	c.484_486del3	31	f		2419	22,1	yes	1				1, liver							prominent iron in kupffer cells	M
Lim BCMD 2008	V162del	c.484_486del3	65	m	15,2	4559		yes	0		0,80	52,1		neg						grade 3 siderosis in hepatocytes and prominent in kupffer cells	H
Pelucchi ClinGent 08	V72F	c.214G>T	58	m	15,7	1091	81	yes	2	0	2,7	160						regular drinker		largely hepatocytes but also kupffer cells	H
Pelucchi ClinGent 08	V72F	c.214G>T	48	m	15,4	956	73	yes	3		2,5	120			yes		chol/TG	1		largely hepatocytes but also kupffer cells	H
Rivard BJH 03	Y64N	c.190T>C	56	m		1759	96	yes	1											Iron deposition hepatocytes and kupffer cells	H
Rivard BJH 03	Y64N	c.190T>C	24	m		647	98	yes	0				1, liver		yes					Iron deposition hepatocytes and kupffer cells	H
Rivard BJH 03	Y64N	c.190T>C	30	f		176	47	no	0											Iron deposition hepatocytes and kupffer cells	H
Cunat ClinChem 2007	G490S	c.1468G>A	71	f		2710	83	unknown	0			350								yes	
Cunat ClinChem 2007	G490S	c.1468G>A	24	m		1262	32	unknown	0											yes	
Cunat ClinChem 2007	R88G	c.262A>G	41	m		2200		yes	1			65								mixed1	M
Cunat ClinChem 2007	5' UTR	c.[-59_-45del]	73	m		1400	80		0			240								yes	
Griffith	R489K	c.1466G>A	54	m		3400		yes	0	0										grade 1 hepatocytes strong kupffer cell	H
Letocart BJH 2009	Y501C	c.1502A>G	35	m	15,3	642	94	no	0				1, liver no splee	neg				neg		significant hepatocellular iron overload, although kupffer cell was also evident	H
